# Saponin constituents of Achyranthes root

**DOI:** 10.1007/s11418-021-01591-1

**Published:** 2022-01-04

**Authors:** Fumiyuki Kiuchi

**Affiliations:** Faculty of Pharmacy, Keio University, 1-5-30 Shibakoen, Minato-ku, Tokyo, 105-8512 Japan

**Keywords:** Achyranthes root, *Achyranthes bidentata*, *Achyranthes fauriei*, Oleanane saponin, Achyranthoside, Betavulgaroside

## Abstract

Achyranthes root is a crude drug used as diuretic, tonic and remedy for blood stasis. Characteristic oleanolic acid saponins with a dicarboxylic acid moiety have been isolated as one of the representative constituents of this crude drug. This review focuses on the triterpene saponin constituents, especially those with a characteristic dicarboxylic acid moiety, of *A. bidentata* and *A. fauriei*. Several groups isolated the saponins and different names were given to one compound in some cases. The names of the compounds are sorted out and the stereochemistry of the dicarboxylic acid moieties are summarized. HPLC analysis of the composition of the saponin constituents and the effect of processing and extraction conditions on the composition are reviewed. Biological activities of the saponin constituents are also summarized.

**Supplementary Information:**

The online version contains supplementary material available at 10.1007/s11418-021-01591-1.

## Introduction

Achyranthes root is a crude drug used as diuretic, tonic, and remedy for blood stasis. In the Japanese Pharmacopoeia, the origin of this crude drug is defined as the root of *Achyranthes fauriei* Leveille et Vaniot or *A. bidentata* Blume (Amaranthaceae) [1]. However, in the Plants of the World Online [2], *A. fauriei* is one of the 19 synonyms of *A. bidentata*. A comprehensive review, covering literatures published up to 2016, of the two medicinal species of *Achyranthes*, *A. bidentata* and *A. aspera*, on the traditional uses, phytochemistry, and pharmacological activities has been published [3]. Phytoecdysones, triterpene saponins, polysaccharides, and polypeptides are the four major classes of the bioactive constituents of Achyranthes root. Phytoecdysones are the first bioactive constituents isolated from *A. fauriei* [4, 5], and the triterpene saponins have characteristic dicarboxylic acid substituents. In recent years, more attention has been paid to the high molecular weight constituents of Achyranthes root, i.e. polypeptides and polysaccharides, in relation to the biological activities [3]. This review focuses on the triterpene saponin constituents, especially those with a characteristic dicarboxylic acid moiety, of *A. bidentata* and *A. fauriei*.

## Isolation and characterization of saponin constituents

**Fig. 1:**
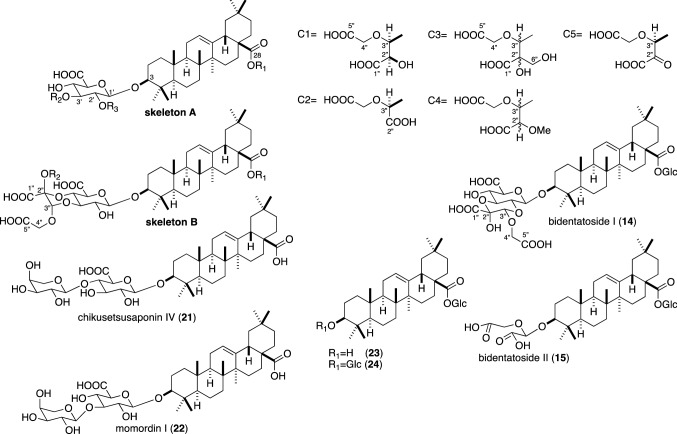
Structures of the saponins isolated from Achyranthes root and related compounds. C-3″ of the dicarboxylic acid moieties C1–C5 attaches to C-3′ of the glucuronic acid moiety of skeleton A through an ether linkage. C-2″ of C5 also forms a hemiacetal linkage to C-4′ of the glucuronic acid moiety to form skeleton B

Achyranthes root contains characteristic oleanane saponins with dicarboxylic acid substituents. These saponins have been isolated by different groups and, in some cases, different names were given to one compound. The structures and names of the saponins are summarized in Fig. 1 and Table 1. In the following text, original names described in the literature are used and, if necessary, the names listed in the first column of Table 1 are followed in parenthesis. The ^13^C NMR data of the saponins with dicarboxylic acid moiety reported in the literatures are summarized in Table S1 (Supplementary Material).

**Table 1:** Summary of the saponins isolated from Achyranthes root and related compounds

Compound	Other names	Skeleton	R_1_	R_2_	R_3_	References
Chikusetsusaponin IVa (**1**)		A	Glc	H	H	[6]
Chikusetsusaponin V (**2**)	Ginsenoside Ro	A	Glc	H	Glc	[6, 19, 22]
Pseudoginsenoside RT_1_ (**3**)		A	Glc	H	Xyl	[6]
28-Desglucosyl- Chikusetsusaponin V (**4**)	Zingibroside R_1_	A	H	H	Glc	[6, 22]
Achyranthoside A (**5**)		B	Glc	Me		[7]
Achyranthoside B (**6**)	Betavulgaroside I Achyranthoside III	B	Glc	H		[7, 11, 16, 20, 24]
Achyranthoside C (**7**)	Betavulgaroside III	A	Glc	C1	H	[8, 10, 11, 22, 23, 25]
Achyranthoside D (**8**)	Betavulgaroside V Achyranthoside I	A	Glc	C1	Glc	[8, 10, 11, 15, 22, 24]
Achyranthoside E (**9**)	Spinacoside C	A	Glc	C2	H	[9, 14, 20, 23, 25]
Achyranthoside F (**10**)		A	Glc	C3	H	[9]
Achyranthoside G (**11**)	Achyranthoside IV	A	H	C1	Glc	[10, 16, 21, 25]
Achyranthoside H (**12**)		A	Glc	C4	H	[10]
Betavulgaroside IV (**13**)	Achyranthoside II	A	H	C1	H	[11, 15, 25]
Bidentatoside I (**14**)						[17]
Bidentatoside II (**15**)						[18]
Momordin Ib (**16**)		A	H	H	H	[20]
Sulfachyranthoside B (**17**)		B	Glc-4-*O*-SO_3_H	H		[24]
Sulfachyranthoside D (**18**)		A	Glc-4-*O*-SO_3_H	C1	Glc	[24]
Betavulgaroside II (**19**)		B	H	H		[11, 25]
Spinacoside D (**20**)		A	H	C2	H	[14]
Momordin I (**22**)		A	H	Ara	H	[28]

A, B, C1–C5: see Fig. 1

*Ara* α-l-arabinopyranosyl, *Glc* β-d-glucopyranosyl, Glc-4-*O*-SO_3_H 4-*O*-sulfooxy-β-d-glucopyranosyl, *Xyl* β-d-xylopyranosyl

Four triterpene saponins were first isolated and characterized from the BuOH soluble fraction of the MeOH extract of Achyranthes root (the root of *A. fauriei*) by Ida et al. [6]. They treated the BuOH soluble fraction with diazomethane and isolated methyl esters of chikusetsusaponins (CSs) IVa (**1**) and V (**2**), pseudoginsenoside RT_1_ (**3**), and 28-desglucosylchikusetsusaponin V (**4**). These compounds are oleanane saponins with glucuronic acid at C-3. From the same source, Ida et al. [7–10] also isolated oleanane saponins, named achyranthosides (ASs) A–H as their methyl esters, which have characteristic dicarboxylic acids bound through an acetal linkage to the glucuronic acid moiety at C-3 of oleanolic acid (Fig. 1). Among these saponins, ASs A (**5**) and B (**6**) have a 1,4-dioxane ring structure formed by two acetal linkages between the dicarboxylic acid substituent and C-3′ and C-4′ of the glucuronic acid [7]. AS A (**5**) is considered to be an artefact formed from AS B (**6**) in the isolation process. In the meanwhile, Yoshikawa et al. [11–13] isolated ASs B (**6**), C (**7**) and D (**8**) as their free carboxylic acid form from the water and MeOH extracts of sugar beet (*Beta vulgaris*, Amaranthaceae), and named betavulgarosides (BVSs) I, III and V, respectively. AS E (**9**) was also isolated from the water extract of spinach (*Spinacia oleracea*, Amaranthaceae) and named spinacoside (SS) C by Yoshikawa’s group [14]. Later, ASs B (**6**) and D (**8**) were isolated from the BuOH soluble fraction of the 70% MeOH extract of *A. bidentata* by a Chinese group and named achyranthosides III and I, respectively [15, 16]. They also isolated a saponin named ASs II (**13**) and IV (**11**) [16], whose structures were identical with those of BVS IV previously isolated from *B. vulgaris* by Yoshikawa et al. [11] and AS G, respectively. Similar oleanane saponins with a characteristic dicarboxylic acid moiety were also isolated from the BuOH soluble fraction of the MeOH extract of *A. bidentata* by a French group and named bidentatosides (BDSs) I (**14**) and II (**15**) [17, 18]. BDS I (**14**) has a dioxane ring similar to that of AS B (**6**). However, the positions of the acetal linkages are not C-3′ and C-4′ but C-2′ and C-3′ of the glucuronic acid moiety [17]. BDS II (**15**) lacks the glucuronic acid moiety, and the dicarboxylic acid attaches directly to the C-3 of oleanolic acid. They also isolated three oleanolic acid saponins without dicarboxylic acid moiety [oleanolic acid-28-*O*-β-d-glucopyranoside (**23**), CS V (**2**), and 3-*O*-β-d-glucopyranosyl-oleanolic acid-28-*O-*β-d-glucopyranoside (**24**)] from the roots of *A. bidentata* [19]. Momordin Ib (**16**) was also isolated from the MeOH extract of *A. bidentata* together with ASs B (**6**) and E (**9**), and CSs IVa (**1**) and V (**2**), and their methyl esters [20]. Methyl ester of 28-desglucosylachyranthoside D (= AS G, **11**) was also isolated from the MeOH extract of *A. bidentata* [21], and ASs C (**7**) and D (**8**) together with CS IV (**21**), ginsenoside Ro (= CS V, **2**), and zingibroside R_1_ (= 28-desglucosyl CS V, **4**) were isolated from the BuOH soluble fraction of the 70% EtOH extract of *A. bidentata* [22]. Methyl and butyl esters of ASs C (**7**) and E (**9**) were also isolated from the BuOH soluble fraction of the MeOH extract of *A. bidentata* [23]. Two achyranthoside derivatives, named sulfachyranthosides (SASs) B (**17**) and D (**18**), which were sulfated at the C-4′″ of the glucose moiety at C-28 of oleanolic acid, together with AS D (**8**) were isolated from a water extract of Achyranthes root [24], and ASs C (**7**), E (**9**), G (**11**), and BVSs II (**19**) and IV (**13**) were identified from the water extract of Achyranthes root by the same group [25]. The plant sources of achyranthoside derivatives are limited. The other plants include *Basella rubra* (Basellaceae) for BVS I (= AS B, **6**) and SS C (= AS E, **9**) [26], and *Pisonia umbellifera* (Nyctaginaceae) for AS E (**9**) and SS D (**20**) [27].

## Stereochemistry of the dicarboxylic acid moiety

**Fig. 2:**
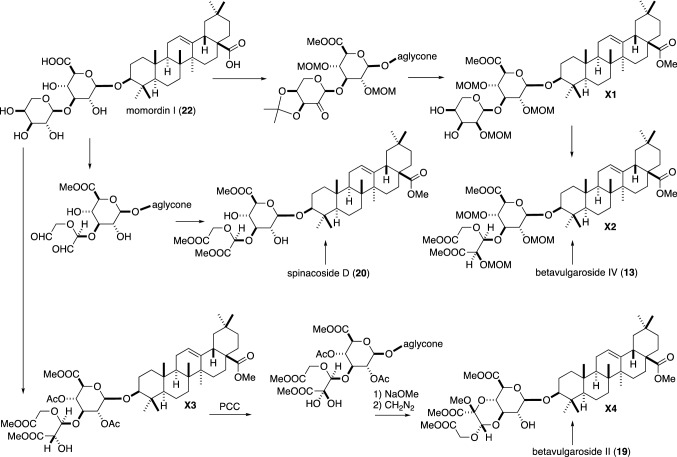
Chemical correlations of the saponins with momordin I

Five types of the dicarboxylic acid moiety (C1–C5 in Fig. 1) have been reported for the saponins isolated from Achyranthes root. Dicarboxylic acid C5 forms the 1,4-dioxane ring of the skeleton B and BDS I (**14**) with the glucuronic acid moiety (Fig. 1). The stereochemistry of AS B (**6**) was established by an X-ray crystallographic analysis of its 28-desglucosyl derivative as shown in Fig. 1 [7]. Yoshikawa et al. established the absolute stereostructures of BVSs III (= AS C, **7**) and IV (**13**) by chemical correlations to momordin I (**22**) (Fig. 2). The α-l-arabinosyl moiety of **22** [28] was converted to α-l-ribosyl derivative (**X1**) whose *cis* diol was then oxidized to give compound **X2**, which was identical with the compound derived from BVS IV (**13**) [29, 30]. Thus, the stereochemistry of the dicarboxylic acid moiety C1 was determined to be [2″*R*, 3″*S*] as shown in Fig. 1. They also oxidized the hydroxy group of C1 of the derivative **X3** to yield **X4**, which was identical with the compound obtained by a treatment of BVS II (**19**) with diazomethane [30]. This established the stereochemistry of the dicarboxylic acid moiety C5 and the stereochemistry of the dioxane ring, which was in agreement with that of AS B (**6**) established by the X-ray crystallographic analysis [7]. The stereochemistry of the dicarboxylic acid moiety C2 of SSs C (= AS E, **9**) and D (**20**) was also determined by a chemical correlation to **22** (Fig. 2) [14]. The stereochemistry of BVS III (= AS C, **7**) was also confirmed by synthesis starting from oleanolic acid, d-glucose and l-arabinose by Zhu et al. [31]. They synthesized BVS III (**7**) and its C-2″ epimer from the same starting materials and showed that the natural C1 moiety could be derived by oxidative cleavage of 3,4-*cis*-diol of not the α-l-arabinopyranosyl derivative but corresponding α-l-ribopyranosyl derivative. Thus, the dicarboxylic acid moiety of ASs B (**6**), C (= BVS III, **7**) and E (= SS C, **9**), BVS IV (**13**), and SS D (**20**) have a common stereochemistry of [3″*S*]. As the dicarboxylic acid part of AS B (C5 in Fig. 1) is an oxidized form of the dicarboxylic acid moiety C1, and the dicarboxylic acid moiety C2 can be formed oxidative decarboxylation of C5 [32], these results suggested that C1, C2, and C5 have a common origin.

**Fig. 3:**
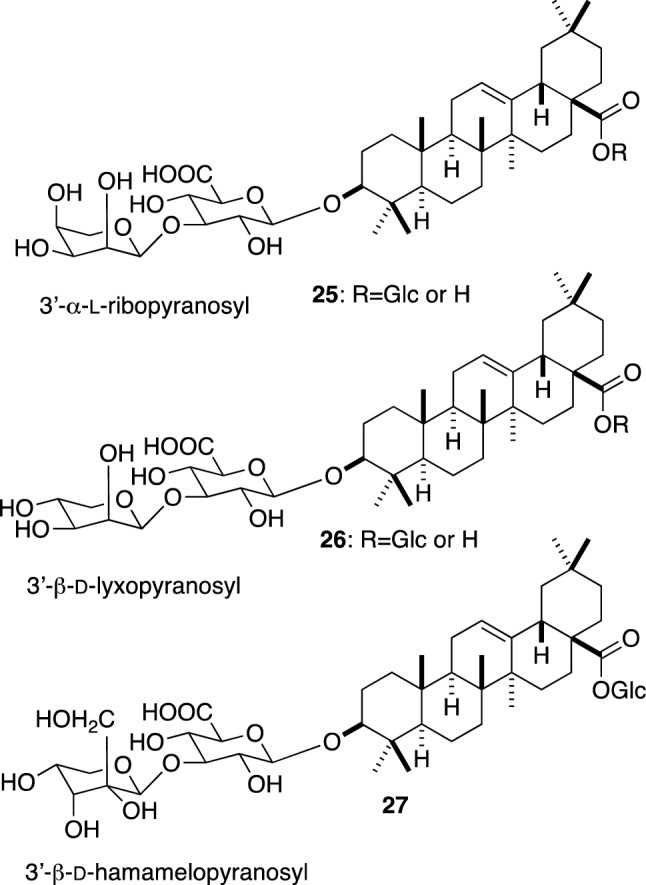
Plausible candidates for the precursor of achyranthosides

As suggested by Yoshikawa et al. [30] and Zhu et al. [31], the dicarboxylic acid moiety can be derived by an oxidative fragmentation of a terminal monosaccharide unit. As described above, the dicarboxylic acid moieties C1, C2 and C5 were correlated to α-l-ribopyranosyl group. However, as l-ribose has not been found in nature, 3′-α-l-ribopyranosyl derivatives **25** are not possible (Fig. 3). Plausible candidates are β-d-lyxopyranosides **26**, which have the required stereochemistry at C-1 and C-2 corresponding to the C-3″ and C-2″ of the dicarboxylic acid moiety, respectively. However, such glycosides have not been detected in Achyranthes root. The dicarboxylic acid moiety C3 of achyranthoside F (**10**) contains an additional hydroxymethyl group at the 2″-position of C1. As the stereochemistry of C3 has not been determined, a derivative of d-hamamelose (2-*C*-hydroxymethylribose) **27** is a possible precursor. However, if the stereochemistry of C3 is the same with that of C1, the terminal sugar moiety should be its enantiomer. As only a limited number of the saponins with such dicarboxylic acid moiety has been reported, a specific pathway seems to be involved in the biosynthesis of the dicarboxylic acid moieties.

## HPLC analysis of saponins

Li et al. [33] reported an HPLC method to analyze main phytoecdysones and triterpenoids in the root of *Achyrantes bidentata*. Four ecdysterones, oleanolic acid and six derivatives of oleanolic acid with glucuronic acid moiety at C-3 (**1** and its ethyl ester, **2**, **4** and its butyl ester, **16**) were identified using an ODS column and a solvent system containing formic acid. Qualification and quantification method for eight triterpenoids (**1** and its ethyl ester, **2**, **4**, **16** and its butyl ester, **23**, and oleanolic acid) of Achyranthes root by HPLC with evaporative light scattering detection and ESI–MS detection was also reported [34]. However, achyranthosides were not detected in these reports.

**Fig. 4:**
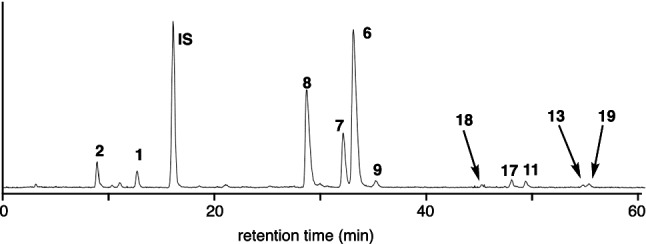
Selected ion monitoring (SIM) chromatogram of a decoction of Achyranthes root

Li et al. [35] reported an LC–MS/MS method to analyze and characterize saponins in *Achyranthes bidentata*, using an ODS column with a solvent system containing formic acid. With this method, 22 oleanane-type triterpenoid saponins including ASs C (**7**) and D (**8**) were characterized. However, the peaks of achyranthosides showed considerable tailing under this condition. The peak shapes of achyranthosides were greatly improved by the use of cationic ion-pair reagents with reversed-phase column, and an LC–MS based quantification method of achyranthosides using a combination of dihexyl ammonium acetate and phenyl-hexylated silica gel column was reported (Fig. 4) [25]. Under this condition, the retention time of the saponins was dependent on the number and position of acidic groups in each molecule. CSs (**1** and **2**), which have only one carboxylic acid of glucuronic acid, eluted first. ASs B–E (**6**–**9**) with three carboxylic acids eluted next, followed by SASs B and D (**17** and **18**) which have an additional sulfonic acid on the glucose moiety at C-28 of oleanolic acid. AS G (**11**), and BVSs II (**19**) and IV (**13**) having four carboxylic acids, one of which is the C-28 of oleanolic acid, showed longer retention times.

## Saponin patterns of Achyranthes root and the effect of different extraction and preparation conditions on the amounts of the saponins

**Fig. 5:**
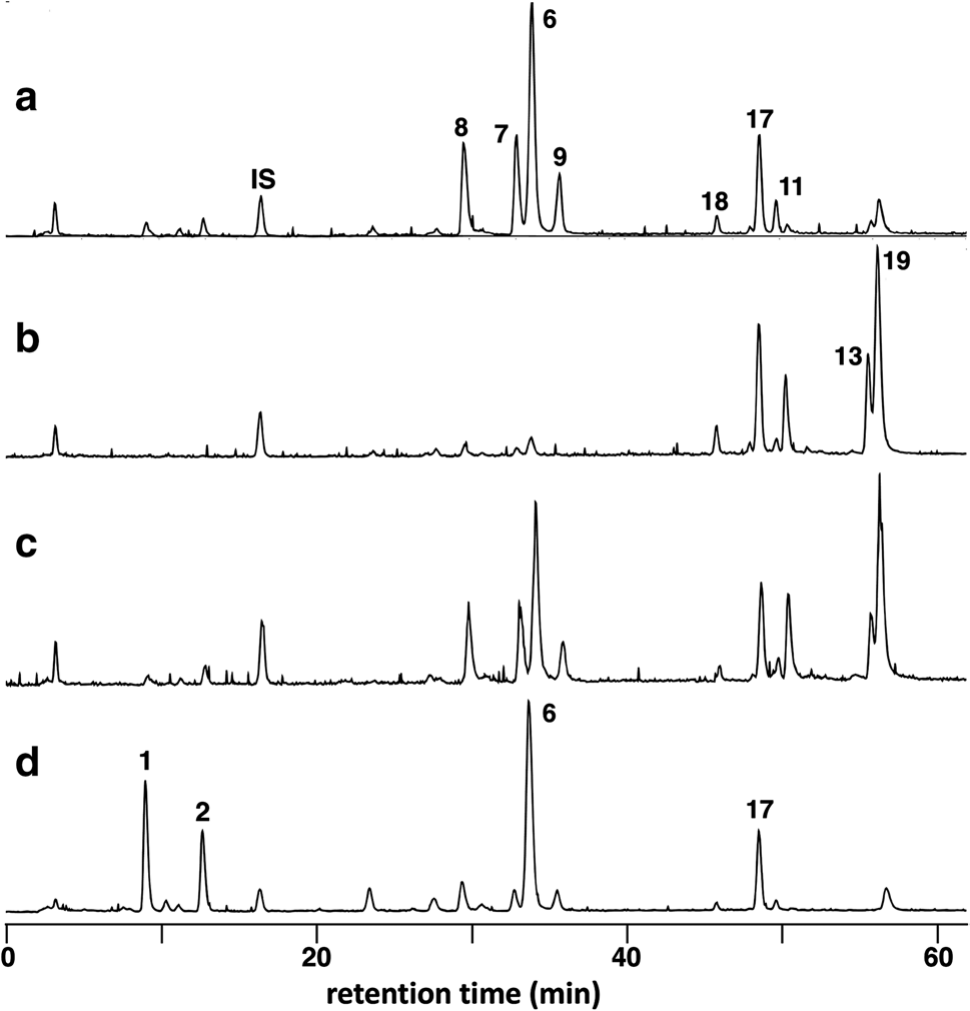
Representative saponin patterns of Achyranthes root in the Japanese market. **a** Saponins with glucose moiety at C-28 are the major constituents; **b** saponins without glucose moiety at C-28 are the major constituents; **c** a mixed pattern of **a** and **b**; **d** a pattern of samples after long storage

Achyranthes roots in the Japanese market showed variable saponin patterns [32]. In an LC–MS analysis by selective ion monitoring of **11** saponins (**1**, **2**, **6**–**9**, **11**, **13**, **17**–**19**), three patterns of saponin composition were observed for the water extracts: (1) the saponins with sugar moiety at C-28 [ASs B (**6**), C (**7**) and D (**8**)] were the major constituents (Fig. 5a), (2) the saponins without sugar moiety at C-28 [BVSs II (**19**) and IV (**13**)] were the major constituents (Fig. 5b), and 3) mixtures of these saponins (Fig. 5c) [32]. In these samples, the amounts of CSs IVa (**1**) and V (**2**) were very small or negligible. However, in some samples stored for a long period and their color changed to dark, CSs IVa (**1**) and V (**2**), and AS B (**6**) were the major constituents (Fig. 5d).

**Fig. 6:**
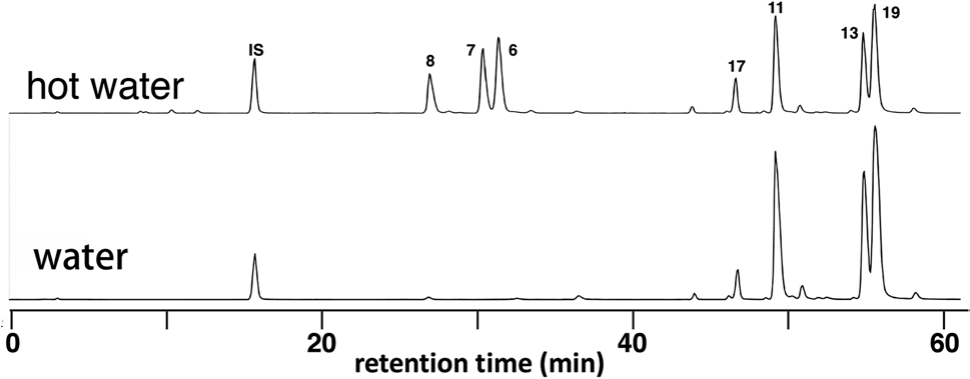
Comparison of the water and hot water extracts of the same crude drug

When hot water was used for the extraction, ASs B (**6**), C (**7**) and D (**8**) were detected even from the samples whose water extract did not contain these saponins (Fig. 6). This was attributed to inactivation of endogenous esterase which hydrolyze the ester linkage at C-28 [32].

Fuchino et al. [36] investigated the effect of drying temperature of the root on the saponin constituents by LC-NMR/MS. The pattern of the saponin constituents varied depending on the drying temperature. Chikusetsusaponins were the major constituents of the samples dried above 70 °C, and from the roots dried at 100 °C for 3 days, they identified CSs IVa (**1**) and V (**2**), 28-desglucosyl CS V (**4**), and oleanolic acid 3-*O*-glucuronide (= momordin Ib, **16**). On the contrary, achyranthosides were detected from the roots dried below 50 °C [36]. Ultra-performance liquid chromatography coupled with quadrupole time-of-fright MS/MS (UPLC-QTOF-MS/MS) analysis was used for monitoring the effect of sulfur-fumigation of Achyranthes root [37]. The amounts of BVSs II (**19**), III (= AS C, **7**) and IV (= AS G, **11**) decreased to 28%, 38% and 37% of the original amounts, respectively, after 2 h of heavy sulfur-fumigation (the weight ratio of sulfur to herbal material = 1:20).

The amounts of the saponins in the extract were variable depending on the extraction conditions. The amounts of the saponins prepared by three extraction methods: water extraction for 24 h, decoction for 30 min, and reflux for 3 h, of the same crude drug sample were compared [25]. AS B (**6**) and D (**8**) were the major saponins and the amounts of CSs IVa (**1**) and V (**2**) were negligible in the water extract. The amounts of AS B (**6**), C (**7**) and D (**8**), especially that of AS B (**6**), increased and small amounts of CS IVa (**1**) and V (**2**) were detected in the decoction. When the sample was extracted under reflux, the amounts of CS IVa (**1**) and V (**2**) increased greatly and that of AS B (**6**) also increased, whereas the amounts of the other saponins were similar to those in the decoction. The results suggested that CS IVa (**1**) and V (**2**) were mainly formed from AS C (**7**) and D (**8**), respectively. On the other hand, the relative amount of AS B (**6**) among achyranthosides increased on prolonged heating, because the dicarboxylic acid moiety of AS B (**6**) forms a six-membered ring structure and more stable compared to the dicarboxylic acid moiety of the other achyranthosides. The amounts of BVS II (**19**) and IV (**13**) in decoction were largely decreased compared with those in the water extract prepared from the same sample. As large amounts of these saponins were found in the precipitates formed by heating of the water extract, these saponins were seemed to precipitate out under heating [32]. When saponins were extracted with BuOH from the water extract, the relative amounts of highly polar saponins, AS D (**8**) and SASs B (**17**) and D (**18**), in the BuOH extract decreased. In addition, when reagent grade BuOH was used, oxidative decarboxylation of AS B (**6**) and BVS II (**19**) occurred resulting in formation of AS E (**9**) and SS D (**20**), respectively. As these changes were not observed with HPLC grade BuOH, which contains not more than 5 ppm of peroxide impurities, the change was attributable to the peroxide impurities contained in the reagent grade BuOH [32].

Localization of the saponin constituents were examined by Jaiswal et al. They separated root samples to cortex, medullary rays and tertiary vascular bundles and analyzed each part to show that, although saponins were detected in all the parts, the amount was highest in cortex followed by medullary rays and tertiary vascular bundles [38]. The contents of oleanolic acid in the acid hydrolysate of the root, stem and leaf of *A. bidentata* were also investigated [39]. All the three parts contained oleanolic acid, and the contents (%) were the highest in early August (root, *ca*. 8%; stem and leaf, *ca*. 4%). In early September, the amount in the root dropped dramatically (*ca*. 1%), whereas the decrease in the stem and leaf was small (*ca*. 3%). In October and November, the content was almost constant in the root (*ca*. 3%) and the stem (*ca*. 1%).

## Biological activity of the saponins of Achyranthes root

Oleanane saponins are one of the major constituents of Achyranthes root and some biological activities have been reported for the saponins. Yoshikawa et al. [11] reported hypoglycemic effect of BVSs II (**15**), III (= AC C, **7**) and IV (**13**). A fraction composed of ASs E (**9**) and F (**10**) prepared from Achyranthes root potently inhibited the interaction between polymorphonuclear leukocytes (PMNs) and E-selectin [9]. AS H (**12**) methyl ester showed strong antiproliferative effect against human breast cancer cells with induction of apoptosis, whereas the effect of methyl esters of ASs C (**7**) and E (**9**) was moderate [40]. A saponin fraction prepared from the BuOH soluble fraction of the 70% EtOH extract of Achyranthes root suppressed IL-1β-induced apoptosis and nuclear factor κB activation in rat chondrocytes [41]. Methyl and butyl esters of oleanolic acid saponins including ASs A (**5**), C (**7**), D (**8**), and E (**9**), and CSs IVa (**1**) and V (**2**) isolated from the BuOH soluble fraction of the MeOH extract of *A. bidentata* were reported to inhibit the formation of osteoclast-like multinucleated cells induced by 1α, 25-dihydroxyvitamin D_3_ [23]. Saponins of *A. bidentata* were also reported to promote osteogenic differentiation of bone marrow stromal cells through the ERK MAPK signaling pathway [42]. Ginsenoside Ro (= CS V, **2**) inhibited adhesion, migration and invasion of colon cancer cells HT29 [43]. The cytotoxicity of *A. fauriei* roots increased by heat processing and the cytotoxic principle against human hepatoma SK-Hep-1 cells was identified as CS IVa (**1**) [44]. The amounts of these compounds also increased in salt-processed Achyranthes root and contributed to the protective effect against LPS-induced acute kidney injury [45].

## Conclusion

Triterpene saponins are one of the major constituents of Achyranthes root. Several groups isolated characteristic saponins with a dicarboxylic acid group and, in some cases, different names were given to one compound. This review sorted out all the triterpene saponins with dicarboxylic acid moiety isolated from Achyranthes root and clarified their relationships. Although the dicarboxylic acid moiety of the saponins has been postulated to be formed by oxidative cleavage of a terminal monosaccharide unit, actual precursor and reactions involved in the biosynthesis remain to be investigated. In recent years, more attention has been paid to the high molecular weight constituents of Achyranthes root in relation to the biological activities. For example, effects of the polypeptides on nervous system [46, 47] and effects of the polysaccharides on bone metabolism [48, 49] have been reported. Nevertheless, saponins are one of the representative constituents of Achyranthes root, and although some biological activities have been reported for the saponins, further investigations on the saponins will be necessary to understand the medicinal property of Achyranthes root and use the crude drug effectively.

## Supplementary Information

Below is the link to the electronic supplementary material.

Supplementary file1 (PDF 56 KB)
