# Supplementary material for: Saponin constituents of Achyranthes root
Source: J Nat Med. 2022 Jan 4;76(2):343–51. doi: 10.1007/s11418-021-01591-1 (PMC8858921; doi:10.1007/s11418-021-01591-1)
Supplement: Supplementary file 1 — Supplementary file1 (PDF 56 KB) [file 11418_2021_1591_MOESM1_ESM.pdf]

Table S1 Reported ^{13}C NMR data of the oleanolic acid saponins with a dicarboxylic acid moiety isolated from Achyranthes root and the same compounds isolated form other plant sources

name[1]	AS A Me ester	BVS I	AS III	AS A (AS B)	AS B	AS B Me ester	BVS III	AS C Me ester	AS C diMe ester	BVS V	AS I	AS D	AS D Me ester	SS C	AS E	AS E Me ester	AS E diMe ester	AS F Me ester	AS IV	AS G	AS G Me ester	AS G Me ester	AS H Me ester	BVS IV	AS II	BDS I	BDS II	SAS B	SAS D	BVS II	SS D
structure[2]	**5a**	**6**	**6**	**6**	**6**	**6a**	**7**	**7a**	**7b**	**8**	**8**	**8**	**8a**	**9**	**9**	**9a**	**9b**	**10a**	**11**	**11**	**11a**	**11a**	**12a**	**13**	**13**	**14**	**15**	**17**	**18**	**19**	**20**
solvent[3]	a	a	a	b	a	a	a	a	a	a	a	a	a	a	b	a	a	a	a	a	a	a	a	a	a	c	d	a	a	a	a
ref.	7	11	16	20	24	7	11	10	23	11	15	24	10	14	20	9	23	9	16	25	10	21	10	11	15	17	18	24	24	11	14
oleanoloc acid																															
1	39.1	38.4	38.6	37.9	38.4	39.0	38.6	38.6	38.6	38.6	38.6	38.6	38.6	38.6	38.1		38.6		38.5	38.5	38.5	38.6	38.6	38.6	38.5	39.3	38.1	38.6	38.7	38.5	38.6
2	27.0	26.4	26.5	25.4	26.4	27.1	26.6	26.5	26.6	26.4	26.4	26.4	26.5	26.6	25.7		26.5		26.5	26.2	26.3	26.3	26.5	26.6	26.6	26.6	25.4	26.6	26.5	26.5	26.6
3	89.5	89.2	89.3	89.6	89.2	90.1	89.2	89.3	89.2	89.5	89.5	89.6	89.3	89.3	89.1	89.4[4]	89.2	89.3[4]	89.3	89.5	89.5	89.5	89.2	89.2	89.1	91.6	88.5	89.4	89.7	89.3	89.3
4	39.9	39.3	39.4	38.6	39.3	40.1	39.5	39.5	39.5	39.5	39.5	39.5	39.5	39.5	38.8		39.4		39.5	39.5	39.5	39.5	39.4	39.5	39.5	39.5	38.5	39.5	39.6	39.5	39.6
5	56.1	55.5	55.6	55.1	55.5	56.2	55.7	55.7	55.7	55.8	55.7	55.8	55.7	55.7	55.2		55.6		55.6	55.7	55.7	55.8	55.7	55.7	55.7	56.6	55.0	55.7	55.8	55.7	55.8
6	19.0	18.3	18.4	17.7	18.3	19.0	18.5	18.4	18.5	18.5	18.4	18.5	18.4	18.5	16.8		18.4		18.4	18.4	18.4	18.5	18.4	18.4	18.4	19.1	17.6	18.5	18.5	18.4	18.5
7	33.6	33.0	33.0	32.5	33.0	33.7	33.1	33.1	33.1	33.1	33.1	33.1	33.1	33.1	32.4		33.1		33.1	33.1	32.9	33.2	33.0	33.1	33.1	33.1	32.0	33.2	33.1	33.3	33.2
8	40.3	39.7	39.8	39.1	39.7	40.2	39.9	39.9	39.9	39.9	39.8	39.9	39.9	39.9	39.2		39.8		39.7	39.6	39.6	39.7	39.9	39.7	39.7	40.0	38.9	39.9	39.9	39.7	39.8
9	48.4	47.8	47.9	47.2	47.8	48.7	48.0	48.0	48.0	48.0	47.9	48.0	48.0	48.0	47.3		48.0		47.9	47.9	47.8	48.0	48.0	48.0	48.7	48.2	46.9	48.0	48.0	47.9	48.0
10	37.4	36.7	36.8	36.1	36.7	37.4	36.9	36.9	36.9	36.9	36.8	36.9	36.9	36.9	36.3		36.9		36.9	36.9	36.8	36.9	36.9	36.9	36.9	37.1	36.1	36.9	36.9	36.9	37.0
11	23.9	23.2	23.3	22.6	23.2	24.3	23.4	23.4	23.4	23.4	23.3	23.4	23.8	23.4	22.8		23.4		23.7	23.8	23.6	23.7	23.7	23.4	23.6	24.3	22.3	23.3	23.3	23.8	23.8
12	122.8	122.7	122.8	122.2	122.7	122.8	122.8	122.8	122.9	122.8	122.8	122.9	122.8	122.8	122.4		122.8		122.5	122.5	122.8	122.5	122.8	122.5	122.5	123.4	122.0	122.9	122.9	122.5	122.3
13	144.0	144.0	144.1	143.5	144.0	144.8	144.1	144.1	144.1	144.1	144.0	144.1	144.1	144.1	143.6		144.1		144.8	144.8	144.1	144.8	144.1	144.8	144.8	144.5	143.4	144.1	144.1	144.8	144.9
14	42.5	42.0	42.0	41.4	42.0	42.6	42.1	42.1	42.1	42.1	42.1	42.1	42.1	42.1	41.1		42.1		41.9	42.1	41.8	42.0	42.1	42.2	42.1	42.4	41.0	42.1	42.1	42.1	42.2
15	28.7	28.1	28.2	27.4	28.1	28.8	28.2	28.2	28.1	28.2	28.2	28.2	28.1	28.3	29.2		28.2		28.0	28.0	28.0	28.3	28.2	28.3	28.1	28.1	27.3	28.2	28.2	28.3	28.4
16	24.3	23.6	23.7	23.1	23.6	24.3	23.7	23.7	24.0	23.6	23.7	23.7	23.4	23.8	23.2		23.7		23.7	23.7	23.3	23.7	23.4	23.8	23.7	24.2	24.1	23.7	23.7	23.8	23.8
17	47.4	46.8	46.9	46.5	46.8	47.1	47.0	47.0	47.0	47.0	46.9	47.0	47.0	47.0	46.6		46.9		46.6	46.7	46.9	46.5	47.0	46.7	46.6	47.0	45.8	47.0	47.0	46.6	46.7
18	42.2	41.6	41.6	41.0	41.6	42.4	41.7	41.7	41.7	41.7	41.7	41.7	41.7	41.7	41.5		41.7		42.1	41.9	41.7	42.1	41.7	42.0	41.9	42.5	40.5	41.7	41.7	42.0	42.1
19	46.6	46.0	46.1	30.0	46.0	46.9	46.2	46.2	46.2	46.2	46.1	46.2	46.2	46.2	30.1		46.1		46.4	46.4	46.0	46.7	46.2	46.4	46.4	46.5	45.1	46.2	46.2	46.4	46.6
20	31.2	30.6	30.7	30.0	30.6	31.4	30.7	30.8	30.8	30.7	30.7	30.8	30.8	30.8	30.1		30.8		30.9	30.9	30.8	31.0	30.8	31.0	30.9	31.0	30.2	30.8	30.8	31.0	31.0
21	34.5	33.8	33.9	33.2	33.8	34.7	34.0	34.0	34.0	34.0	33.9	34.0	34.0	34.0	33.4		34.0		34.2	34.2	33.9	34.3	34.0	34.2	34.2	34.0	33.0	34.0	34.0	34.2	34.3
22	33.0	32.4	32.5	32.3	32.4	33.6	32.5	32.5	32.5	32.5	32.5	32.5	32.5	32.6	32.5		32.5		33.2	33.2	32.7	33.2	32.5	32.1	33.2	34.2	31.5	32.5	32.5	33.2	33.2
23	28.6	27.9	28.0	27.4	27.9	28.2	28.1	28.1	28.1	28.0	28.0	28.0	28.2	28.1	27.7		28.1		28.3	28.3	28.0	28.1	28.1	28.2	28.3	27.8	27.8	28.1	28.0	28.1	28.2
24	17.4	16.7	16.8	16.1	16.7	16.9	16.9	16.8	16.9	16.7	16.6	16.7	16.7	16.9	16.4		16.8		16.8	16.7	16.7	16.7	16.8	16.9	16.9	16.4	16.5	16.8	16.7	16.9	17.0
25	16.0	15.3	15.4	14.8	15.3	16.1	15.5	15.5	15.5	15.5	15.4	15.5	15.5	15.5	14.9		15.5		15.4	15.4	15.4	15.4	15.5	15.4	15.4	15.6	15.1	15.5	15.5	15.4	15.5
26	18.0	17.3	17.4	16.7	17.3	17.9	17.4	17.4	17.5	17.4	17.4	17.4	17.5	17.5	17.9		17.4		17.3	17.3	17.1	17.4	17.4	17.4	17.3	17.3	17.1	17.4	17.4	17.3	17.4
27	26.6	26.0	26.0	25.4	26.0	26.7	26.1	26.1	26.1	26.1	26.0	26.1	26.1	26.1	27.6		26.1		26.2	26.1	26.1	26.2	26.1	26.2	26.2	25.9	25.9	26.1	26.1	26.2	26.2
28	176.1	176.3	176.4	177.0	176.3	179.9	176.4	176.4	176.4	176.4	176.4	176.4	176.4	176.4	176.5	176.4	176.4	176.4	180.1	180.1	178.0	180.1	176.4	180.2	180.1	178.7	175.6	176.4	176.4	180.1	180.1
29	33.6	33.0	33.1	32.5	33.0	33.7	32.1	33.1	33.1	33.1	33.1	33.1	33.1	33.1	32.6		33.0		33.3	33.3	33.1	33.3	33.1	33.1	33.3	33.2	32.6	33.2	33.1	33.3	33.2
30	24.1	23.5	23.6	23.0	23.5	24.3	23.6	23.7	23.7	23.4	23.6	23.6	23.6	23.6	23.1		23.6		23.7	23.8	23.6	23.8	23.6	23.8	23.7	24.1	23.5	23.6	23.6	23.8	23.8
28-OMe																					52.1	–									
C-3-GluA																															
1’	107.5	107.4	107.5	105.2	107.4	107.5	106.7	106.7	106.7	105.1	105.1	105.2	104.9	106.9	105.1	106.8	106.8	106.6	107.6	105.2	105.1	105.1	106.8	106.8	106.8	106.3	–	107.6	105.2	107.6	106.8
2’	71.9	71.9	72.0	71.1	71.9	72.0	74.8	74.5	74.1	78.3	78.2	78.3	78.3	75.0	76.2	74.9	74.9	74.7	72.1	78.3	78.2	78.3	74.8	74.8	74.8	72.2	–	72.1	78.2	72.1	75.0
3’	71.8	72.4	72.5	71.4	72.4	72.4	85.4	84.7	85.1	83.9	83.6	83.8	82.6	85.7	86.9	84.3	84.7	84.9	72.6	83.9	82.4	82.5	84.4	85.5	85.4	73.1	–	72.6	83.5	72.5	85.6
4’	69.6	70.0	70.1	70.2	70.0	69.9	72.3	71.8	72.5	72.9	72.8	72.9	72.3	71.9	71.6	71.2	71.6	71.8	70.2	72.4	72.4	72.5	71.6	72.4	72.4	71.8	–	70.2	73.0	70.1	71.9
5’	73.7	75.1	75.1	73.5	75.1	74.3	77.5	76.8	77.1	77.2	77.2	77.3	76.5	77.6	78.0	76.8	77.1	76.7	75.2	76.4	76.6	76.6	76.7	77.6	77.6	78.0	–	75.3	77.3	75.3	77.6
6’	168.5	171.4	171.7	173.9	171.4	168.8	172.4	170.2	172.4	172.2	172.2	172.3	170.0	172.4	174.6	170.3	172.5	170.2	171.7	172.3	170.1	170.1	170.3	172.4	172.4	175.9	–	171.9	172.4	171.6	172.3
6'-OMe	52.8					51.2		51.7	–				51.8			52.5	–	52.1			51.6	52.1	51.8								
dicarboxylic acid[5]	C5-3,4a	C5-3,4a	C5-3,4a	C5-3,4a	C5-3,4a	C5-3,4a	C1	C1	C1	C1	C1	C1	C1	C2	C2	C2	C2	C3	C1	C1	C1	C1	C4	C1	C1	C5-2,3a	C2	C5-3,4a	C1	C5-3,4a	C2
1”	166.6	171.0	171.3	176.4	171.0	169.6	174.8	172.4	172.4	174.5	174.5	174.6	172.3	–	–	–	–	172.5	171.3	176.0	172.3	172.3	170.3	174.6	174.8	174.5	–	171.4	174.7	171.2	–
2”	97.5	93.8	93.9	94.4	93.8	93.9	74.2	73.8	74.1	73.0	72.9	72.9	72.5	171.7	176.4	168.1	168.2	81.8	94.0	72.9	72.6	72.3	82.7	74.2	72.6	100.1	171.0	94.1	72.8	94.0	171.9
3”	96.9	97.9	98.0	96.3	97.9	97.9	105.4	104.6	104.7	105.2	105.1	105.2	104.1	101.2	102.4	99.5	99.5	104.9	98.1	105.1	104.0	104.1	103.2	105.4	105.4	96.8	100.7	98.1	105.1	98.1	101.2
4”	64.4	64.7	64.9	67.0	64.7	64.6	65.1	63.9	64.0	65.7	65.7	65.7	64.4	64.7	68.1	63.2	63.0	64.7	64.9	64.8	64.3	64.1	63.6	64.3	65.1	67.3	69.5	64.9	65.8	64.9	65.0
5”	169.4	172.2	172.3	180.0	172.2	169.0	173.9	171.1	171.3	173.7	173.7	173.7	171.0	173.5	176.9	170.6	170.7	171.2	172.3	172.4	171.0	171.0	170.9	173.9	173.9	177.1	173.0	172.4	173.8	172.3	173.5
6”																		74.3													
1”-OMe	52.7					52.5		52.1	51.7				52.0			–		52.1			51.8	51.8	52.1								
2”-OMe	51.4					–		–	–				–			52.1	51.8	–			–	–	58.8								
5”-OMe	51.9					52.4		51.3	51.7				51.3			51.8	51.5	51.3			51.3	51.3	51.3								
6''-OMe	–					–		–	–				–			–		59.5			–	–	–								
C-28-Glc																															
1'''	95.9	95.7	95.7	94.6	95.8	95.9	95.7	95.7	95.7	95.7	95.7	95.8	95.8	95.8	94.9	95.8	95.7	95.7					95.7			95.5	94.0	95.1	95.2		
2'''	74.4	74.0	74.0	72.5	74.2	77.4	74.1	74.0	74.1	74.1	74.1	74.2	74.2	74.1	74.2	74.2	74.1	74.1					74.1			73.2	72.3	74.4	74.4		
3'''	79.1	78.7	78.8	76.8	79.0	79.1	78.8	78.8	79.3	78.8	78.8	78.9	78.9	78.9	78.1	79.0	79.3	78.9					78.9			77.4	76.5	77.6	77.6		
4'''	71.4	70.9	71.0	69.7	71.1	71.4	71.1	71.0	71.1	71.1	71.0	71.1	71.1	71.1	71.6	71.1	71.0	71.1					71.1			70.6	69.5	75.9	75.9		
5'''	79.5	79.1	79.2	77.5	79.4	79.5	79.3	79.2	78.9	79.2	79.3	79.3	79.3	79.3	77.3	79.3	78.9	79.3					79.3			78.0	77.6	77.2	77.2		
6'''	62.6	62.0	62.1	60.9	62.2	62.6	62.2	62.1	62.2	62.2	62.1	62.2	62.2	62.2	61.3	62.3	62.1	62.2					62.2			61.4	60.5	62.0	61.9		
2’-Glc																															
1''''										103.6	103.6	103.7	103.6							103.7	103.6	103.6							103.6		
2''''										76.3	76.3	76.4	76.2							76.3	76.2	76.2							76.3		
3''''										78.1	77.9	78.1	78.2							78.1	78.1	78.2							78.1		
4''''										72.4	72.4	72.4	72.5							72.4	72.3	72.5							72.4		
5''''										77.8	78.0	78.0	78.1							78.2	78.2	78.2							78.1		
6''''										63.2	63.1	63.2	63.1							63.2	63.1	63.2							63.2		

1) names in the literature. AS: achyranthoside, BVS: betavulgaroside, SS: spinacoside, BDS: bidentatoside

2) **5a**–**12a**: trimethyl ester, **7b** and **9b**: dimethyl ester

3) a: C_5D_5N, b: $C_5D_5N:H_2O$=4:1, c: CD_3OD, d: DMSO-d_6

4) Chemical shifts of aglycone part of each compound were not reported except for C-3 and C-28.

5) C5-3,4a: dioxane ring formation with 3'- and 4'- of glucuronic acid, C5-2,3a: dioxane ring formation with 2'- and 3'- of glucuronic acid.
